# A Moderated Mediation Model Linking Excessive Enterprise Social Media Usage With Job Performance

**DOI:** 10.3389/fpsyg.2022.884946

**Published:** 2022-05-13

**Authors:** Haowen Li, Muhammad Ali, Muhammad Waqas Amin, Haoshen Liang

**Affiliations:** ^1^School of Management and Economics, North China University of Water Resources and Electric Power, Zhengzhou, China; ^2^Department of Business Administration, Federal Urdu University of Arts, Science, and Technology, Islamabad, Pakistan; ^3^Shool of Management, Yanshan University, Qinhuangdao, China; ^4^College of Business, Technische Universität Dresden, Dresden, Germany

**Keywords:** excessive ESM usage, ESM usage regret, ESM usage inertia, COVID-19 threat, employee performance

## Abstract

Despite the larger interest of information systems scholars in excessive ESM usage, little is known about how excessive ESM usage is related to employee performance. This study focused on excessive ESM usage and investigated its impact on employee performance. Based on the *status quo* perspective with the integration of social cognitive theory, this study first proposed that excessive ESM usage has a positive and negative relationship with employee performance through ESM usage regret and ESM usage inertia. Furthermore, COVID-19 threat moderates the direct relationship between excessive ESM usage and ESM usage regret, and ESM usage inertia. Time-lagged, multi-source data collected in China support most of our hypothesis. Results reveal that excessive ESM has a positive and negative indirect effect on employee performance via ESM usage regret and ESM usage inertia. Furthermore, the COVID-19 threat moderates the positive direct effect of excessive ESM usage on ESM usage inertia. In the later section, theoretical contributions and practical implications are discussed.

## Introduction

In recent years the outbreak of the Corona Virus Disease 2019 (COVID-19) has imposed a significant threat to public health. Resultantly, governments have implemented strategies to cope with the COVID-19 such as social distancing, lockdowns (Khan, 2021). Essentially following the government policies, organizations adopted a work from home or outstation approach for employees to perform their duties. Enterprise social media (ESM) gained much attention from organizational scholars due to its effectiveness in facilitating organizational strategies (Ngai et al., 2015). In the current time of global crises, ESM has enabled organizations to support marketing agendas, increase collaboration among employees, and enhance innovation (Tussyadiah and Zach, 2013; Ali et al., 2020a; Islam et al., 2020). Besides these benefits, ESM is considered a double-edged sword (Bao et al., 2020), that is, it also brings negative impacts on employees (Zahrai et al., 2022b), such as decreased performance (Cao and Yu, 2019). Organizations are eager to gain social media adaptation benefits and avoid negative consequences. Many scholars have focused on investigating methods and suggested strategies to increase enterprise social media usage benefits. However, such investigations seem insufficient, provided that recently Sun et al. (2021) noted that ESM adoption does not bring intended benefits to the organizations. ESM adoption is a strategic decision that has significant financial and social costs for the organizations. Therefore, to avoid certain unintended costs, scholars have called for more research to better understand methods and devise strategies for organizations to increase the benefits of ESM adoption (Cao et al., 2021; Islam et al., 2021; Yousaf et al., 2022). These previous research trends and calls underscore the need to further investigate how ESM improves or constrains the performance of employees.

In order to achieve the goal of this study to answer the above calls, we develop our model of ESM impact on employee performance based on information systems literature. Research has proposed the *status quo* as a factor to elaborate on social media usage and its implications (Furneaux and Wade, 2011; Nel and Boshoff, 2020; Wang J. et al., 2021). *Status quo* refers to an equilibrium between positive and negative factors describing a situation (Lewin, 1947). Hence, the *status quo* can describe the usage of ESM and its consequences including positive and negative. In particular, this approach can elaborate on factors that facilitate and constrain employee performance using ESM. For instance, their study highlighted the need to adopt a balanced approach to explore factors that explain the positive side of social media (enablers) and that explain the negative side of social media (inhibitors). Accordingly, in our study, we adopt a balanced approach to exploring the implications of ESM from an enablers and inhibitors perspective.

In view of performance enablers, scholars have generally focused on knowledge creation (Kao and Wu, 2016), absorptive capacity (Ali and Bahadur, 2019), and social capital (Sun et al., 2019). Away from such a dominant perspective of performance enablers of ESM, we focus on a specific unpleasant emotional experience (i.e., regret) (Yuen et al., 2022). We propose that regret is a powerful motivator that enables employees to go against the *status quo* and put efforts to increase performance. Regret is a negative emotion that develops when an individual encounters their bad decision (Cao and Sun, 2018). In others, regrets emerge when individuals perceive that they have made a wrong decision to engage in a situation (Kaur et al., 2016). Nevertheless, research has noted that when individuals encounter regret, they try to shape their behavior to undo the decision that affects their future actions (Islam et al., 2019). According to this view, ESM users who experience regret may avoid extensively engaging in social media usage, rather divert their energies to perform their job tasks. Thus, this study focus on ESM usage regret as an enabler of employee performance who excessively use ESM.

In view of performance inhibitors, studies on social media have dominantly focused on emotional exhaustion, fatigue, technostress, cyberbullying (Schneider et al., 2017; Dhir et al., 2018; Wang Y. et al., 2021). Though we recognize the value these studies have made in understanding the performance inhibitors of ESM. We extend this line by investigating why individuals persist excessively using ESM from a *status quo* perspective. This perspective is useful to explain why individuals keep on excessively using ESM despite the benefits of changing their usage behavior patterns (Wang J. et al., 2021). Thus, *status quo* persistence might provide a useful framework to understand performance inhibitors. We propose that such persistence of ESM usage can be explained by ESM usage inertia (Jung et al., 2019). Bravo and Ostos (2021) show that ESM usage inertia is an effective source that significantly affects social media user behavior. Accordingly, we identify ESM usage inertia as an effective *status quo* experience of employees that keeps them persistent in the excessive use of ESM and decreases their employee performance.

In addition, this research further investigates the boundary condition under which direct impacts of excessive ESM usage on ESM usage regret, and ESM usage inertia are strengthened or weakened. As research *status quo* perspective noted that positive and negative evaluation of the situation is a cognitive process (Li et al., 2016). We integrate insights from social cognitive theory which suggests that environmental factors significantly affect the cognitive evaluation of the situation and individual behavioral reaction (Bandura, 1986). COVID-19 is a significant environmental condition significantly employees’ behavior toward technology usage (Naeem and Ozuem, 2021). Islam et al. (2021) noted that due to COVID-19 people are more willing to interact and communicate using social media. Accordingly, we propose that COVID-19 threat is an environmental condition that represents the extent to which employees perceive the threat to get infected with COVID-19 (Borah et al., 2022). When confronted with excessive ESM usage, employees with high perceived COVID-19 threat might feel less ESM usage regret, whereas their feeling to maintain the *status quo* of excessive ESM usage (inertia) might be high. Hence, we propose a buffering moderating role of COVID-19 threat in the relationship between excessive ESM usage and ESM usage regret and a strengthening moderating role of COVID-19 threat in the relationship between excessive ESM usage and ESM usage inertia.

**FIGURE 1:**
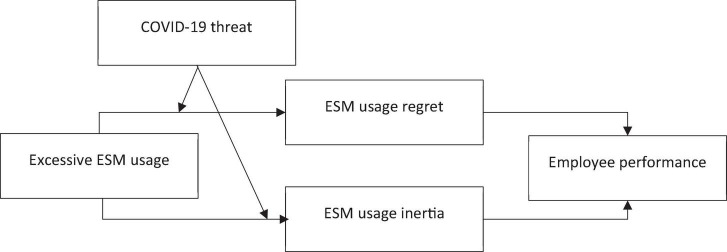
Conceptual model of the study.

In summary, this study develops a model based on the *status quo* perspective in information systems literature (Furneaux and Wade, 2011; Wang J. et al., 2021) to investigate performance enablers, and performance inhibitors of excessive ESM users (Figure 1 shows the conceptual model of this study). In doing so, this study contributes to the literature in the following ways. First, this study contributes to the literature on social media by investigating two mechanisms through which excessive ESM use is related to employee performance. Second, this study contributes to the *status quo* literature by integrating *status quo* with ESM literature to investigate how ESM usage regret improves employee performance, while ESM usage inertia constrains employee performance who excessively use social media. Finally, this study contributes to the literature by investigating moderating role of the COVID-19 threat that affects the relationship between excessive ESM usage and ESM usage regret, and ESM usage inertia.

## Literature Review and Hypotheses Development

### Theoretical Background

A *status quo* perspective is an approach in information systems that focuses to examine user behavior by considering enablers and inhibitors as two factors that affect usage decisions and their consequences. Enablers provide positive assessments of technology that facilitate behavioral aims, whereas inhibitors are negative assessments of the technology *status quo* that obstruct those same intentions (Wei et al., 2020; Yousaf et al., 2022). Previous research has established the *status quo* perspective’s effectiveness in comprehending users’ IT decision-making and its consequences. For instance, their study noted that ESM usage regret is a negative response to technology use (inhibitor) which may increase employee performance (Bao et al., 2020). On the other hand, inertia is found to be an enabler that increases social media usage intentions (Wang J. et al., 2021) which eventually might lead to decreased employee performance.

Research on the *status quo* in social media is generally applied to user intentions to use social media. The literature on two factors of the *status quo* perspective reveals that discontinuance intentions were explained by the positive impacts of behavioral-change drivers (enablers) and the negative effects that motivate the continuation of usage (inhibitors) (Wang J. et al., 2021). In our context of excessive ESM usage, we believe that when employees experience performance enablers (ESM usage regret) they are likely to decrease excessive ESM usage therefore, they would have more time to focus on their tasks, therefore, increasing employee performance. On the other, when employees experience inertia, they are likely to value excessive ESM usage, leading to decrease employee performance. In this line, this research applies existing findings and logic of the two factors approach to the *status quo* and highlights the dual mechanism through excessive ESM usage is linked with employee performance.

In addition, research on *status quo* perspectives is limited in the way that it does not describe conditions that lead to a positive or negative evaluation of their decision (Nel and Boshoff, 2020; Wang J. et al., 2021). To further enhance the *status quo* perspective, we integrate the lens of social cognitive theory (Bandura, 1986). This theory states that environmental factors play a significant role in the evaluation process of the personal factors (i.e., decisions), that eventually lead to the emotional and behavioral outcome of the evaluation process (Cao et al., 2019). In recent years, the COVID-19 outbreak has evolved as an important environmental concern in social, societal, and professional settings. COVID-19 has changed the way of social interactions, communication, businesses, and education (Hu et al., 2020). For instance, due to the threat that individuals can get infected by physical interaction with others, people are more involved in using technologies, i.e., social media to interact and communicate with each other (Bennett et al., 2021). This way, we identify COVID-19 threat as an important boundary condition that may alter the effect of individual evaluation of their decision and actions to maintain or change the *status quo*. In other words, we propose that COVID-19 threat would present an important boundary condition that can alter the impact of excessive ESM usage, and thus, ESM usage regret, and ESM usage inertia.

### Excessive Enterprise Social Media Usage and Regret

There has been a lot of interest in looking into regret in different domains, resulting in different definitions for this notion. For instance, according to Bell (1982), regret implies that people make decisions based on their evaluations of options, which are influenced by the worth and likelihood of the chosen outcome, as well as the amount of regret for missed opportunities. As a result, according to the above view, regret is a rational reaction to an unfavorable comparison between the consequences of chosen and foregone alternatives. On the other hand, psychologists view regret as a negative emotion that people try to avoid (Tsiros, 1998). Accordingly, in psychology, regret is a negative cognitive-emotional experience when individuals feel their current situation could have been different and more favorable (Towers et al., 2016). According to the above views and conceptualizations, regret is an affective reaction to the comparison process. In other words, when people compare and discover their decision outcomes as unfavorable, they perceive that other decision choices could have made the situation favorable. Furthermore, regret indicates a self-blame because of wrong personal decision. In this sense, regret diverts the attention of individuals to the situation that resulted in unfavorable decision outcomes. A critical aspect of regret is that it makes individuals consider other decision options and opportunities (Joseph-Williams et al., 2011). As such, regret represents a relationship between choices and emotions.

In the context of our study, we propose that excessive ESM usage indicates a large amount of information to handle (Zhang et al., 2020; Zahrai et al., 2022a), which is likely to cause regret. For instance, one basic need to use ESM is to improve communication, interaction, and information exchange among different employees. In this sense, ESM provides access to other users’ information and facilitates network connections among employees. However, as the number of social network connections increases, the employees have to interact with more connections. Moreover, employees are likely to receive and process more information. Such ESM activities might distract employees’ focus from their job-related tasks. Thus, hurting their performance which may result in employees’ regret. Thus, we propose excessive ESM usage is likely to generate a difference between employees’ ideal outcome and actual outcome related to their work resulting in a negative outcome e.g., regret.

In their study, Dhir et al. (2019) noted that negative outcomes of social media use triggers regret. More precisely, in their study with a Chinese sample, Cao and Sun (2018) studied the negative impact of social media-related overloads (information, communication, and social overload). They proposed that regret is a consequence of such social media overloads. Another key factor related to ESM usage is that individual users receive requests to provide support (Maier et al., 2014). Employees can request social support from their virtual friends because ESM networking platforms provide easy and efficient social interactions with other individuals. Through, reciprocity, individual employees are also can request virtual support from their online colleagues. Yet, individual employees may be confronted with an increasing number of virtual support requests as social networks get wider due to excessive ESM usage. However, with limited resources (i.e., time and energy), EMS users must provide some sort of help due to the rule of reciprocity in social connections (Maier et al., 2014). When the ESM requests more virtual support than the individual is capable of providing, the network may be overburdened, leading to a sense of general loss of control over the social situation resulting in a feeling of regret. Thus, we propose that:

H1: *Excessive ESM usage is positively related to ESM usage regret.*

### Excessive Enterprise Social Media Usage and Inertia

According to Polites and Karahanna (2012), inertia is a user’s persistent attachment to existing usage patterns even there are available better alternative options or incentives. As such, inertia indicates an individual’s willingness to follow the *status quo*. According to the above definition of inertia, in our context of ESM usage, inertia is an employee’s attachment to and persistence in excessive ESM usage. In this view, inertia indicates a biased user behavior toward continuous usage patterns of ESM. Previous studies have noted that changing user behavior is not without cost. For instance, changing ESM usage patterns can cause stress, and generate a feeling of resource loss (Tandon et al., 2021), thereby demotivating employees to change from their current excessive ESM usage patterns. Accordingly, instead of changing from current excessive ESM usage, employees may persist in maintaining the *status quo*.

Research has suggested that inertia has behavioral, cognitive, and affective components. In this line, the research found that habit and affective commitment are key determinants of inertia (Polites and Karahanna, 2012; Sun et al., 2017). Based on these findings, and taking view from the *status quo* perspective, we propose that excessive ESM usage indicates a habit and affective commitment that leads to developing ESM usage inertia. In our context of ESM usage, habit is an automated behavior of using ESM (Limayem et al., 2007). This automated behavior is motivated by environmental cues. Research has suggested that when individuals continuously engage in a certain behavior (excessive ESM usage) they do not optimally measure the costs of their decision (Polites and Karahanna, 2012). In this way, excessive ESM usage motivates employees to maintain the *status quo* by continuously using the ESM in the same pattern, resulting in ESM usage inertia. Furthermore, when employees excessively use ESM, they are able to generate more connections. However, changing the patterns may generate a feeling of resource loss (friends’ support) (Dhir et al., 2018). Therefore, employees may seek to refrain from changing the *status quo* by keeping on using ESM excessively. Together, excessive ESM usage is likely to influence EMS usage inertia. Therefore, we propose the following hypothesis:

H2: *Excessive ESM usage is positively related to ESM usage inertia.*

### Regret and Employee Performance

Regret is a cognitive psychological state that arises as a result of a negative comparison between the outcomes of chosen and foregone options (Connolly and Zeelenberg, 2002). A strong desire to undo or reverse the decision that resulted in the regrettable outcomes will result from such a reaction (Zeelenberg and Pieters, 2007). In the context of this study, we propose that such a negative comparison of the situation, i.e., the performance of employees excessively using ESM may realize that such behavior is affecting job performance, and the decision to decrease ESM usage would lead to increase job performance than the earlier decision of excessive ESM usage. With such a comparison of current and potential performance situations due to excessive ESM usage, an employee will feel regret and would be willing to spend more time on performing job tasks rather than excessively using ESM. In this way, an employee may like to change the *status quo* of excessive ESM usage.

Furthermore, research has noted that regret as a negative emotion can direct individual behavior (Towers et al., 2016). For example, regret as a situation aversive emotion triggers a situation in which individuals decide to change their regret experience by adjusting their behavior (Zeelenberg and Pieters, 2007). In this way, we believe that employees who experience regret due to excessive ESM usage would engage in regret experience management. As a result, they would focus their attention on performing their job task rather than spending more time on excessive ESM usage. The above mechanism has received indirect support from several empirical studies. For instance, Kang et al. (2013) noted that regret will result in low continuance intention. Moreover, Chang et al. (2014) found that regret motivates users to change their social media usage patterns. Therefore, this study proposes that when employees experience ESM usage regret due to low performance resulting from excessive ESM usage, they are likely to alter the feeling of regret by focusing on job tasks and lowering ESM usage. Thus, we propose the following hypothesis:

H3: *ESM usage regret is positively related to employee performance.*

### Inertia and Employee Performance

Because of their cognitive limitations, individuals may be resistant to changing the *status quo* out of inertia (Lee and Joshi, 2017). In the context of ESM, inertia refers to a bias toward an established behavioral pattern in which individuals continue to excessively utilize an ESM despite the presence of incentives to switch (Polites and Karahanna, 2012). In terms of excessive ESM usage, because passive users rely on existing usage patterns to influence subsequent choices, they are less likely to change the current service (Lin et al., 2015). Employees try to maintain and extend their existing network because their network connections give them social, cognitive, and psychological support which supports employee performance (Soda et al., 2021). As such, ESM can afford these resources to individual users. Therefore, employees might believe that decreasing ESM usage can damage the key resources needed to effectively perform their job. As such, ESM usage inertia would prevent excessive ESM users to go against the *status quo*. Therefore, we propose that ESM usage inertia is likely to constrain employees to improve their ESM usage behavior. Thus, we propose that inertia will motivate employees to maintain the *status quo* of excessive ESM usage leading to a decline in employee performance. Thus, ESM usage inertia would negatively affect employee performance. Thus, we develop the following hypothesis:

H4: *ESM usage inertia is negatively related to employee performance.*

### The Mediating Role of Enterprise Social Media Usage Regret and Enterprise Social Media Usage Inertia

Till now we proposed that excessive ESM usage is positively related to ESM usage regret (H1). Further, we proposed that ESM usage regret is positively related to employee performance (H3). Taken together based on the *status quo* perspective, we propose that excessive social media usage is positively related to employee performance via ESM usage regret.

H5: *Excessive ESM usage has an indirect positive impact on employee performance via ESM usage regret.*

Moreover, we proposed that excessive ESM usage is positively related to ESM usage inertia (H2). Further, we proposed that ESM usage inertia is negatively related to employee performance (H4). Taken together based on the *status quo* perspective, we propose that excessive social media usage is negatively related to employee performance via ESM usage inertia.

H6: *Excessive ESM usage has an indirect negative impact on employee performance via ESM usage inertia.*

### The Moderating Role of the Corona Virus Disease 2019 Threat

Till now, we proposed that excessive ESM usage has a positive and negative relationship with employee performance via ESM usage regret and ESM usage inertia. Research on social media usage has stressed contextual factors, indicating that context plays important role in ESM usage behavior. For instance, Islam et al. (2021) noted that how people use online shopping channels is significantly affected by the COVID-19 pandemic. Moreover, Chakraborty et al. (2021) noted that COVID-19 has significantly impacted the behavior of social networking sites users. In line with these studies, we assume that COVID-19 also plays a critical role in how excessive ESM users perceive the *status quo* perspective. Particularly, with the integration of social cognitive theory and *status quo* perspective, we propose that the COVID-19 threat moderates the relationship between excessive ESM usage and ESM usage regret and inertia. COVID-19 threat is defined as the subjective perception of an individual about the objective risk of COVID-19 infection (Raghubir and Latimer, 2014). COVID-19 threat is assumed to substantially impact how individuals perceive ESM usage (Mohammadi, 2015).

COVID-19 is an infectious respiratory disease, which has significant psychological and behavioral effects on employees especially when there is an increase in the rate of infections and fatalities. In many cases, a large portion of employees have to work from home and a small portion of the workforce can work on-site (Khan, 2021). Thus, to perform job-related tasks in a situation where there is a serious health risk, employees are willing to adopt ESM to complete their tasks in a situation where they do not have direct contact with other employees. Research has noted that threat creates anxiety and people try to find ways to tackle the situation (Ye et al., 2019). Accordingly, we expect that with a high COVID-19 threat, employees are more willing to excessively use ESM with low regret because EMS guards against COVID-19 infection.

In this article, we explore the threat posed by COVID-19, which could be the first global health threat. Some people have health problems that make them more susceptible to respiratory infections such as COVID-19, asthma, and immunosuppression (Raghubir and Latimer, 2014). Therefore, in the COVID-19 crisis, to stay safe, these employees need to take special measures to avoid any risky interactions with other people such as by maintaining social distance. Still in order to perform their duties employees need continuous interaction with internal employees and external suppliers and customers. ESM is an important tool that supports employees to effectively perform their work tasks while maintaining social distance to avoid COVID-19 threat (Bennett et al., 2021). Because ESM enables employees to perform their tasks without exposing them to the COVID-19 pandemic. Employees may feel satisfied with their excessive ESM usage behavior because it allows them to interact, communicate, and maintain social relationships with important individuals without health risks. Therefore, according to social cognitive theory, COVID-19 threat is an important environmental factor that affects the behavior and perceptions about excessive ESM usage. Thus, COVID-19 threat can be determined as a moderating condition in the relationship between excessive ESM usage behavior and ESM usage regret and inertia. Accordingly, we propose that when there is a high risk of COVID-19, individuals feel less regret for excessive ESM usage, and they are more likely to keep using ESM excessively (ESM usage inertia). We offer the following moderation hypotheses:

H7: *COVID-19 threat moderates the effect of Excessive ESM usage on ESM usage regret such that the negative impact is weaker at a high level of COVID-19 threat and vice-versa.*
H8: *COVID-19 threat moderates the effect of Excessive ESM usage on ESM usage inertia such that the positive impact is stronger at a high level of COVID-19 threat and vice-versa.*

## Materials and Methods

### Sample and Data Collection

We followed previous studies on social media to test the conceptual model of this study using a survey methodology to collect data from organizations in cities located in the central region of China (Masood et al., 2020; Shang et al., 2021, 2022; Wang et al., 2022). We used validated measures for all variables from the literature. EMBA students help us identify organizations that are using ESM for interaction and communication among employees and customers. These organizations are using public and private social network platforms. In both cases, the ESM services are adapted according to the needs of the organization. Service providers are liable to maintain the ESM and improve/modify ESM according to the organization’s needs. We first provided a brief introduction to the HR managers of seven organizations and requested the participation of their organizations in the survey study. In response, three organizations agreed to take part in a voluntary survey.

We conducted interviews with random employees to verify they use ESM to communicate and interact with organization members and customers. These organizations are suitable to test the hypotheses of this study because they have adopted ESM for over 5 years. Moreover, they have adopted social distancing to protect their employees. Then, we shared our survey measures with the HR managers and asked if they believe the items clearly explain the purpose of the item. We make a few modifications according to the suggestions of the HR managers.

We requested the HR department to help us distribute the survey questionnaire and collect the responses from the employees and managers. With the help of an HR representative, in phase one we asked employees to answer questions about their social media usage, ESM usage regret, ESM usage inertia, and demographic characteristics, such as age, gender, and education, and their enterprise-related characteristics, such as the length of time since they entered the company, department, frequently used social media, social media friends. In phase two after 1 month of phase one, we shared a questionnaire with the leaders and asked them to rate the performance of their subordinates.

We used Godden’s (2004) method to assess the appropriate sample size to test our model. At a 95% confidence level with a 6% margin of error, assuming a 10,000 population, recommended sample size was 267. We distributed 591 questionnaires to the employees. In response, we received 315 responses. Among the responses, there was 5 incomplete response that we removed in data screening. Collectively, we have a 53% response rate from employees. We received 310 responses from supervisors who rated the performance of their direct subordinates. Among our final sample, there is 61% male, and 39% female. Among respondents, 81% have at least 5 years of job tenure in the organization. Moreover, among respondents, there are 31% from the sales department, 20% from the finance department, 20% from the HR department, and 20% from the manufacturing department. Finally, 80% of the respondents hold at least a bachelor’s degree.

### Survey Measures

The survey measures are based on validated measures in the literature. All the items were measured with a seven-point Likert scale ranging from 1 = strongly disagree to 7 = strongly agree. All survey measures were translated to Chinese using the back-translation approach recommended by Brislin (1980). We used a three-item scale developed by Caplan and High (2006) to measure excessive ESM usage. We adopted three items to measure ESM usage regret. Items for this measure were adopted from Tsiros and Mittal (2000). We used three items to measure inertia adopted from Anderson and Srinivasan (2003). We adopted a scale developed to measure COVID-19 threat Clarke et al. (2021). Employee performance was measured using six items adapted from Kuvaas (2006). Finally, based on the previous studies (Khan and Ali, 2018; Pitafi et al., 2018; Ali and Bahadur, 2019; Ali et al., 2019; Khan et al., 2019; Raza et al., 2020), we controlled the effect of demographic variables which studies have suggested could affect the findings of the empirical analysis.

### Analysis and Results

We adopted a two-step approach to testing the measurement model and structural model of this study (Hair et al., 2011). First, we used AMOS 23.0 to test direct relationships among key variables. Then, to test the mediating and moderating hypotheses, we used PROCESS macro in SPSS. This approach to testing direct hypotheses and mediating and moderating hypotheses using different analytical tools is validated by various scholars (Pitafi et al., 2018; Ali et al., 2019; Cao et al., 2021).

### Measurement Model

Although, in this study multi-source data was collected in multi-phases which is considered appropriate to avoid common method bias (Li et al., 2019; Mehmood et al., 2020). Yet, to further validate that data is not affected by the potential effect of common method bias, we first used the established single factor analysis of Harman (1976). Results of Harman single factor analysis did not reveal any dominant factor, suggesting that common method bias is not a concern in this study. Second, we performed confirmatory factors analysis on hypothesized five factors model and single-factor model in which all constructs to combined into a single variable. We found that hypothesized model provides reasonable fit to data (χ^2^ = 199.82, *df* = 125, CFI = 0.97, GFI = 0.94, and RMSEA = 0.04), and single-factor model provides lower than acceptable fit indices (χ^2^ = 1733.39, *df* = 135, CFI = 0.42, GFI = 0.62, and RMSEA = 0.20). Thus, these results validate that common method bias is not a significant issue in our findings (Bahadur and Ali, 2021). Furthermore, significant moderation effects are not possible with data that is effect by common method bias. In this way, our significant results of moderation analysis also provide further support that common method bias is not a significant issue in our data.

Results of confirmatory factor analysis are reported in Table 1. As reported, loadings of all items are above the acceptable range of 0.54, indicating the distinctiveness of the items. Further, values of composite reliability are above 0.74, providing support for convergent validity of the constructs. As reported in Table 1, values of average variance extracted (AVE) of all constructs are greater than the threshold of 0.50. Furthermore, we calculated the square root of AVE. Furthermore, the values of Cronbach’s alpha are above 0.70. Table 2 presents the mean, standard deviations, the correlations among key constructs, and values of square roots of AVE. The results indicate the square roots of AVE of all constructs are higher than the correlation between constructs. Together, analysis indicates the validity of the measurement model and justifies performing structural model analysis.

**TABLE 1:** Results of confirmatory factor analysis.

Variables	CR	AVE	Cronbach’s alpha	Loadings
EESMU	0.74	0.50	0.74	0.66–0.76
ESMU-regret	0.95	0.87	0.95	0.54–0.85
ESMU-inertia	0.75	0.51	0.75	0.91–0.96
COVID-19 threat	0.76	0.51	0.75	0.64–0.77
Employee performance	0.90	0.59	0.90	0.66–0.83

*N = 310, EESMU = excessive ESM usage.*

**TABLE 2:** Correlation matrix.

Variables	Mean	Standard deviation	EESMU	ESMU-regret	ESMU-inertia	COVID-19 threat	Employee performance
EESMU	3.38	0.79	0.70				
ESMU-Regret	3.41	1.00	0.19**	0.93			
ESMU-Inertia	4.69	0.95	0.27**	0.12*	0.71		
COVID-19 threat	2.97	0.82	0.02	0.07	–0.08	0.71	
Employee performance	3.49	0.45	0.14*	0.30**	−0.11*	0.10	0.77

N = 310, EESMU = excessive ESM usage, square roots of AVE in diagonal cells. *p = 0.05, **p = 0.01.

### Structural Model

**FIGURE 2:**
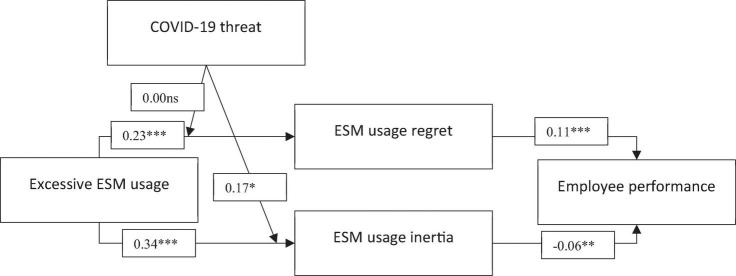
Results of hypotheses test. *p = 0.05, **p = 0.01, ***p = 0.001.

The analysis results of the structural model of this study are reported in Table 3 and Figure 2. We found reasonable fit indices for hypothesized structural model (χ^2^ = 225.55, *df* = 129, CFI = 0.96, GFI = 0.93, and RMSEA = 0.05). As proposed in H1, results show that excessive ESM usage is significantly related to ESM usage regret (β = 0.34, *p* < 0.001). Thus, results provide support for H1. Results also provide support for H2 (β = 0.23, *p* < 0.001). Particularly, results show that excessive ESM usage is significantly related to EMS usage inertia. Moreover, results reveal that ESM usage regret is positively related to employee performance (β = 0.34, *p* < 0.001), supporting H3. In addition, results reveal that ESM usage inertia is negatively related to employee performance (β = 0.34, *p* < 0.001). Thus, the results support H4.

**TABLE 3:** Results of regression analysis.

Independent variables	Dependent variables	Effect	SE	t
EESMU	ESMU-inertia	0.34	0.07	4.95***
EESMU	ESMU-regret	0.23	0.07	3.46***
ESMU-Inertia	Employee performance	–0.06	0.02	−2.62**
ESMU-Regret	Employee performance	0.11	0.03	4.47***
Gender	Employee performance	0.03	0.03	0.94
Age	Employee performance	–0.01	0.02	–0.59
Education	Employee performance	0.01	0.05	0.10
ESMU -Experience	Employee performance	0.04	0.02	1.70
Job tenure	Employee performance	0.00	0.02	–0.14
COVID-19 threat	ESMU-regret	–0.01	0.03	–0.17
Interaction 1	ESMU-regret	0.00	0.04	0.02
COVID-19 threat	ESMU-inertia	–0.07	0.07	–1.12
Interaction 2	ESMU-inertia	0.17	0.08	2.14*

N = 310, EESMU = excessive ESM usage, Interaction 1 = EESMU X COVID-19 threat, Interaction 2 = EESMU X COVID-19 threat. *p = 0.05, **p = 0.01, ***p = 0.001.

To test the mediation hypotheses, we followed previous studies (Ali et al., 2021) and used simple mediation Model 4 of PROCESS macro. Supporting H5, results of the mediation analysis show that excessive ESM usage is positively related to employee performance via ESM usage regret [effect = 0.031, SE = 0.01, 95%CI (0.01, 0.06)]. Furthermore, results reveal that the indirect of excessive ESM usage on employee performance via ESM usage inertia are significantly negative. Thus, these results support H6 [effect = –0.02, SE = 0.01, 95%CI (–0.05, –0.01)].

**FIGURE 3:**
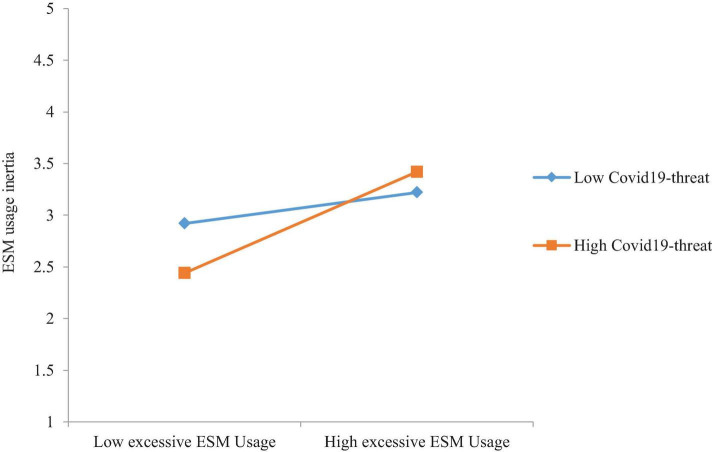
Interactive effect of COVID-19 threat and excessive ESM usage on ESM usage inertia.

In the next step, moderation hypotheses were tested using PROCESS macro Model 1 as applied by early studies (Khan et al., 2019; Ali et al., 2020b). Results indicate that there is no significant direct effect of COVID-19 threat on the relationship between excessive ESM usage and regret (β = 0.01, ns). Thus, the results do not support H7. The results of the H8 analysis reveal that COVID-19 threat significantly moderates the relationship between excessive ESM usage and ESM usage inertia (β = 0.17, *p* < 0.05). We followed previous research to conduct a slop test. Figure 3 shows the moderating role of COVID-19 threat on the relationship between excessive ESM usage and ESM usage inertia. Slop test results reveal that the impact of excessive ESM usage on ESM usage inertia is high, at one standard deviation above the mean level of COVID-19 threat (β = 0.46, *p* < 0.001) compared with when COVID-19 threat is at the mean level (β = 0.32, *p* < 0.05) and when COVID-19 threat is at one standard deviation below mean level (β = 0.17, ns). Thus, results support H8, suggesting that COVID-19 threat strengthens the impact of excessive ESM usage on ESM usage inertia. Collectively, our analysis found support for the conceptual model of this study by providing empirical support for hypothesized relationships.

## Discussion and Contributions

Our study developed and tested a moderated mediation model to highlight existing puzzles surrounding the costs and benefits of using ESM. Specifically, the hypotheses based on the *status quo* perspective in integration with social cognitive theory demonstrated that excessive ESM usage has a dual impact on employee performance via ESM usage inertia and ESM usage regret. More specifically, results reveal that excessive ESM usage has a direct positive effect on ESM usage inertia and ESM usage regret. In turn, ESM usage inertia negatively affects employee performance, whereas ESM usage regret positively affects employee performance. Furthermore, current pandemic situation, the COVID-19 threat moderates the relationship between excessive ESM usage and ESM usage inertia in a way that the positive impact becomes stronger when employees perceive a high COVID-19 threat. However, we did not find a significant moderating effect of COVID-19 threat on the relationship between excessive ESM usage and ESM usage regret. We speculate that there may be other variables such as COVID-19-anxiety that may exercise a stronger moderating effect on the relationship between excessive ESM usage and ESM usage regret. Nevertheless, we expect future studies to investigate other moderating variables which may affect the relationship between excessive ESM usage and ESM usage regret.

### Theoretical Implications

Our study contributes to the literature in the following ways. First, this study investigates psychological mechanisms linking excessive ESM usage with employee performance. Research on information systems adoption reveals that social media is a double-edged sword (Bao et al., 2020), which has positive and negative impacts on employees. Positive or negative consequences depend on user online behavior and contextual factors. Following previous studies, this research provides evidence of the double-edge notion in the context of ESM. Although excessive usage is generally related to negative consequences (Yu et al., 2018; Cao and Yu, 2019), an individual preference for maintaining or changing the *status quo* can change user behavior and outcomes. In this way, our study found that how ESM impacts employee performance is a function of ESM usage decision evaluation and decision about the *status quo*.

Second, this study contributes to the literature by providing two mechanisms (i.e., ESM usage regret, ESM usage inertia) based on the *status quo* perspective. Particularly, ESM usage regrets as a performance enabler predictor of employee performance. In view of performance enablers, scholars have generally focused on knowledge creation (Kao and Wu, 2016), absorptive capacity (Ali and Bahadur, 2019), and social capital (Sun et al., 2019). Research on social media has found regret is a negative emotion that motivates individuals to change social media usage patterns (Islam et al., 2019). Based on this stream of research, we affirm that ESM usage regret motivates employees to avoid excessive ESM usage, rather invest more time and energy on their routine tasks, which we found improves employee performance. Moreover, this study identified ESM usage inertia as a performance inhibitor that is linked with decreased employee performance. Previous research has generally focused on two different lines of mechanisms to investigate performance enablers and performance inhibitors. For instance, in terms of performance inhibitors, research generally focused on emotional exhaustion, fatigue, technostress, cyberbullying (Schneider et al., 2017; Dhir et al., 2018; Wang Y. et al., 2021). The present study reveals that ESM usage inertia is an important mechanism that is linked with decreased employee performance due to excessive ESM usage. Thus, dual mechanism based on *status quo* by simultaneously investigating performance enablers and performance inhibitors, this study enriches our understanding of how excessive ESM usage is related to employee performance.

Third, the identification of COVID-19 threat as a moderator in the relationship between excessive ESM usage and ESM usage regret and ESM usage inertia also contributes to the literature. Our findings show that excessive ESM usage has two paths that are differently linked with ESM usage regret and ESM usage inertia at a high level of COVID-19 threat. Specifically, excessive ESM users are likely to perceive ESM as a positive tool to help them perform their job and maintain a social relationships while avoiding the risk of getting infected by COVID-19, thus, they are more likely to experience inertia. Therefore, we identify and empirically tested that COVID-19 threat as an environmental factor significantly affects employees’ behavior to use ESM. However, our empirical results did not support the moderating role of COVID-19 threat on the relationship between excessive ESM usage and ESM usage regret. This indicates that ESM usage regret is different from ESM usage inertia and factors that elaborate on ESM usage regret need more investigation by future scholars.

### Practical Implications

Our study offers suggestions for practice. First, we found that excessive ESM usage brings both negative and positive evaluations of the situation. For example, we found that ESM usage is positively related to ESM usage regret and ESM usage inertia. These results suggest that managers should devise strategies to prevent users from inertia, however, they should promote standards that motivate employees to evaluate their decision on appropriate ESM usage so that their performance is not affected due to excessive ESM usage. One way to prevent excessive ESM usage is by providing usage training and improved face-to-face interactions so that employees have closer physical interaction to prevent spending excessive time on ESM to find related information to perform their job. Second, COVID-19 has presented a huge threat to individuals and organizations. Moreover, organizations are more willing to adopt social technological tools to facilitate their employees to perform their jobs. However, as a devastating impact of COVID-19, employees may excessively depend on ESM to develop and maintain professional contacts. In such cases, organizations are suggested to adopt plans and devise protocols to avoid excessive ESM usage. One way is to allow a small number of employees to work at different times at the workplace. Nevertheless, excessive ESM usage brings performance drawbacks that need a clear strategy from the organization in the current COVID-19 situation. Finally, this study provides ways to understand that there are different implications of excessive ESM usage by employees, with some being positive and others being negative. We encourage management who encounter excessive ESM usage by employees to curb rationally about how to generate regret and avoid usage inertia as a reaction to excessive ESM usage by employees. Manager’s feedback on ESM usage and performance of employees, with more focus on physical meetings and discussions about work plans and goals, can better shape employees’ ESM usage behavior to improve their performance.

### Limitations and Future Research Directions

This research also acknowledges limitations that provide avenues for future research. First, we collected data from organizations located in cities located in the central region of China. Though, the Chinese sample is considered appropriate to investigate because organizations adopt social media. Yet, China has a unique social and organizational culture. Therefore, we invite future scholars to validate our model in other cultural settings, i.e., Western countries. Second, most of the data come from male members 61%. ESM usage behavior and effect of stress situations (i.e., COVID-19 threat) may vary among the male and female populations. Therefore, our findings may generate biased results. Future studies, therefore, should replicate findings of our results in female-dominant samples. Third, this study adopted the *status quo* perspective to investigate the mediating role of ESM usage regret and ESM usage inertia. Taking the *status quo* perspective, there could be other factors that can mediate the relationship between excessive ESM usage and employee performance, such as guild feeling. Thus, we recommend future studies investigate other mediating factors based on the *status quo* perspective to enrich our understanding of the effects and consequences of excessive ESM usage. Fourth, this study investigated COVID-19 threat as a moderator which has significant implications for the use of technology. Besides, it is possible that by using social media employees receive more news about the COVID-19 pandemic. The news related to COVID-19 can create a devastating impact on employees’ job engagement (Lee et al., 2017). Therefore, we invite future scholars to investigate factors that might have negative implications for ESM usage by employees. Finally, though, we have collected data in two phases. Yet, our independent and mediating variables are measured using the same respondents in phase one. We invite future scholars to adopt advanced methodologies such as daily dairy to provide more validated findings.

## Data Availability Statement

The raw data supporting the conclusions of this article will be made available by the authors, without undue reservation.

## Author Contributions

HWL: conceptualization and revision. MA: conceptualization and drafting. MAm: drafting and analysis. HSL: revision. All authors contributed to the article and approved the submitted version.

## Conflict of Interest

The authors declare that the research was conducted in the absence of any commercial or financial relationships that could be construed as a potential conflict of interest.

## Publisher’s Note

All claims expressed in this article are solely those of the authors and do not necessarily represent those of their affiliated organizations, or those of the publisher, the editors and the reviewers. Any product that may be evaluated in this article, or claim that may be made by its manufacturer, is not guaranteed or endorsed by the publisher.
